# Genetic Characterization of Multidrug-Resistant *E. coli* Isolates from Bloodstream Infections in Lithuania

**DOI:** 10.3390/microorganisms10020449

**Published:** 2022-02-15

**Authors:** Tatjana Kirtikliene, Aistė Mierauskaitė, Ilona Razmienė, Nomeda Kuisiene

**Affiliations:** 1 Department of Microbiology and Biotechnology, Institute of Biosciences, Life Sciences Center, Vilnius University, LT-10257 Vilnius, Lithuania; nomeda.kuisiene@gf.vu.lt; 2 Clinical Testing Department, National Public Health Surveillance Laboratory, LT-10210 Vilnius, Lithuania; aiste.mierauskaite@nvspl.lt (A.M.); ilona.razmiene@yahoo.com (I.R.)

**Keywords:** *E. coli*, virulence factors, genotyping, resistance genes

## Abstract

Extraintestinal pathogenic *Escherichia coli* (ExPEC) isolates are a main cause of bloodstream infections. The aim of this study was to characterize 256 β-lactam–resistant, bacteremia-causing *E. coli* isolates collected from 12 healthcare institutions in Lithuania in 2014 and 2018. All isolates were identified as *E. coli* via MALDI-TOF MS and VITEK ^®^2. In addition, the isolates were analyzed for the presence of 29 resistance genes and 13 virulence genes, divided into phylogenetic groups (A, B1, B2, C, D, E, and F), and characterized using rep-PCR genotyping methods (BOX-PCR and (GTG)_5_-PCR). Analyzing the results of this study showed *tetA-strB-sul2-TEM-NDM-strA-fosA-AIM-sul3-aadA-CTX-M-9* to be the most common resistance gene combination (67.2% of all isolates). Additionally, the most common virulence genes established were *fimH* (98.4% of all isolates), *fyuA* (91.8%), and *traT* (81.3%) and the most common gene combination was *fuyA-fimH-iroN* (58.6% of all isolates). Next, the isolates were separated into four phylogenetic groups: A, B1, B2, and F, where group A isolates were detected at a significantly higher frequency (79.3% of all isolates). Finally, a total of 235 genotyping profiles were established using rep-PCR methods, and all profiles were separated into fourteen genotypic clusters, with each cluster containing profiles with a variety of virulence and resistance genes not restricted to any specific cluster. The results of this study elucidate E. coli antimicrobial resistance patterns by highlighting the variability and diversity of resistance and virulence genes and providing phylogenetic classification, genetic profiling, and clustering data. These results may improve clinical control of multidrug-resistant infections in healthcare institutions and contribute to the prevention of potential outbreaks.

## 1. Introduction

*Escherichia coli* is an opportunistic bacteria, and the most frequently isolated organism in patients with bacteremia [1]. In some cases, *E. coli* infections result in death [2]. *E. coli* strains that induce extraintestinal diseases and survive in the bloodstream are classified as extraintestinal pathogenic *E. coli* (ExPEC) [3]. The most common extraintestinal site colonized by these bacteria is the urinary tract, which is a common source of bloodstream infections [4].

According to the current phylogenetic classification, *E. coli* isolates are divided into seven groups (A, B1, B2, C, D, E, and F) [5]. ExPEC strains generally belong to groups B2 and D, and the commensal strains that survive in the intestines generally belong to groups A or B1 [5].

*E. coli* is resistant to a large group of relevant antimicrobial agents, including aminoglycosides, polymyxins, and broad-spectrum β-lactams. Therefore, all known mechanisms of antibiotic resistance, including enzymatic degradation, altered targets, and active efflux, can be found in *E. coli* isolates. The genes associated with resistance can be located in chromosomes or plasmids. They can be transmitted between isolates or transferred to another *Enterobacteriaceae* species [6]. Moreover, ExPEC strains may have several virulence factors, such as host defense subverting mechanisms, iron acquisition systems, toxins and adhesion, all of which may be important factors in host infection. Furthermore, bacteria without virulence factor coding genes in the bacterial chromosome may acquire them by transmission from bacteria with virulence factor coding genes as a result of these genes usually being located on mobile genetic elements, such as pathogenicity island or plasmids [7]. Several virulence factors may facilitate bacterial cells to infect and colonize the host and have been associated with bloodstream infections [1]. However, predictions of initial severity based on the presence of bacterial virulence factors are not fully accurate [8].

*E. coli* is a major cause of bloodstream infections worldwide. According to previous studies [9], *E. coli* was classified as the most or second-most common pathogen associated with bloodstream infections in Australia, Canada, Denmark, Finland, Iceland, New Zealand, Sweden, and the USA. However, it is less widespread in Lithuania, as the third-most common cause of bacterial infection at healthcare institutions. Moreover, in Lithuania the number of multidrug-resistant bloodstream infections caused by *E. coli* is increasing annually [10]. Therefore, knowledge of virulence factors, antimicrobial resistance determinants, and genotype classifications of bacteremia-causing *E. coli* strains is important for determining the epidemiological profiles of bloodstream infections, which could be beneficial for establishing specific interventions. 

The aim of this study was to characterize multidrug-resistant, bacteremia-causing *E. coli* isolates collected from different healthcare institutions in Lithuania in 2014 and 2018 and compare the mechanisms of spread and pathogenicity observed in 2014 with those observed in 2018.

## 2. Materials and Methods

### 2.1. Collection of Clinical E. coli Isolates, Identification, and Genomic DNA Extraction

A total of 256 β-lactam–resistant *E. coli* isolates were selected from samples collected in 2014 and 2018 from patients with bloodstream infections at 12 different healthcare institutions (I, II, III, IV, V, VI, VII, VIII, IX, X, XI, and XII) in Lithuania (Supplementary Table S1). All isolates were cultivated by hospitals on blood agar for 17–24 h and then sent to National Public Health Surveillance Laboratory for final antibiotic resistance confirmation and pure culture identification using a MALDI-TOF Biotyper (Bruker, Bollerica, MA, USA). Resistance to β-lactam antibiotics was confirmed using the disc diffusion method on Müller–Hinton agar and an automated VITEK ^®^2 system (bioMérieux, Marcy-l’Étoile, France). Antibiotic resistance data were evaluated using the EUCAST *E. coli* breakpoints, and commercial disks of the following antibiotics were used: ampicillin (10 µg), amoxicillin/clavulanic acid (20/10 µg), meropenem (10 µg), cefuroxime (30 µg), amikacin (30 µg), ampicillin/sulbactam (10/10 µg), cefotaxime (5 µg), piperacillin/tazobactam (30/6 µg), ceftazidime (10 µg), ciprofloxacin (5 µg), trimethoprim/sulfamethoxazole (1.25/23.75 µg, gentamicin (10 µg), and imipenem (10 µg). The colistin E-test was also used (BioMaxima, Lublin, Poland). 

To extract genomic DNA, the isolates were cultivated on tryptic soy agar at 30 °C for 12 h. Genomic DNA was extracted using the GeneJET Genomic DNA Purification Kit (Thermo Fisher Scientific, Waltham, MA, USA) according to the manufacturer’s protocol.

### 2.2. Characterization of E. coli Genes Associated with Antibiotic Resistance and Virulence

Genes associated with antibiotic resistance and virulence were detected after the collection and identification of *E. coli* isolates. A total of 29 antibiotic resistance genes and 13 bacterial virulence genes were identified via PCR, using primers reported in previously published studies (Table 1). The PCR reaction mixture had a total volume of 50 µL, which included DreamTaq Green PCR Master Mix (2X) (Thermo Fisher Scientific, Waltham, MA, USA), 0.25 μM of each primer, and 1 µL of E. coli genomic DNA. The PCR conditions were established according to previously published protocols. After PCR analysis, the amplification products were analyzed by 1% agarose gel electrophoresis.

### 2.3. Phylogenetic Classification

To assign *E. coli* isolates to one of the seven main phylogenetic groups, four marker genes (*chuA*, *yjaA*, *TspE4C2*, and *arpA*) and an additional gene (*trpA*) were targeted. Quadruplex PCR was performed as previously described [5,22]. Based on the PCR products, the strains were classified as lineage A, B1, B2, C, D, E, or F.

### 2.4. Genotyping of E. coli Isolates

To analyze the spread of multidrug-resistant *E. coli* clinical isolates in Lithuania, two genotyping methods were used. First, BOX-PCR genotyping was performed. Next, the results were evaluated to identify isolates belonging to the same strain. These isolates were then analyzed via (GTG)5-PCR. BOX-PCR and (GTG)5-PCR were performed according to Dombek et al. [23] and Versalovic et al. [24], respectively. The reaction mixture used for both PCR methods had a total volume of 50 μL, containing DreamTaq Green PCR Master Mix (2X) (Thermo Fisher Scientific, Waltham, MA, USA), 0.5 μM of primer (BOX-PCR: BOXA1R, 5′-CTA CGG CAA GGC GAC GCT GAC G-3′; (GTG)5: 5′-GTGGTGGTGGTGGTG-3′), and 1 µL of bacterial DNA. PCR conditions were established as described by Versalovic et al. [24]. The amplification products were analyzed by 1% agarose gel electrophoresis. 

### 2.5. Data Analysis

Electrophoresis profiles of the BOX-PCR and (GTG)_5_-PCR products were determined using BioNumerics 8.0 software (Applied Maths, Sint-Martens-Latem, Belgium) and Tree Of Life (iTOL) v6, an online tool for phylogenetic tree preparation [25]. Next, dendrograms were constructed using the unweighted-pair group method with the arithmetic mean (UPGMA). 

The χ^2^ test or Fisher’s exact test was used to compare categorical variables. Statistical significance was set at *p* < 0.05. Statistical analyses were performed using SPSS software (version 10.0; SPSS Inc., Chicago, IL, USA).

## 3. Results

### 3.1. Identification and Characterization of E. coli Antibiotic Resistance Genes

In 2014 and 2018, 256 clinical isolates of *E. coli* were collected from 12 different healthcare institutions in Lithuania. All isolates were identified as *E. coli*. Phenotypic resistance to antibiotics was determined according to EUCAST recommendations. All isolates were resistant to one or more third generation cephalosporins and/or one or more carbapenems. Next, the presence of genetic resistance determinants was confirmed in all isolates. The genetic resistance determinants were then divided into groups according to the six types of antibiotic resistance observed in clinical *E. coli* cases: β-lactam resistance, sulfonamide resistance, aminoglycoside resistance, tetracycline resistance, fosfomycin resistance, and polymyxin (colistin) resistance (Table 1). Screening of these gene groups showed that the most common resistance genes were *tetA* (100% of isolates), *strB* (99.2%), *sul2* (99.2%), *TEM* (98.8%), *NDM* (98.4%), *strA* (98.4%), *fosA* (97.3%), *AIM* (97.3%), *sul3* (92.6%), *aadA* (92.6%), and the CTX-M-9 group (90.6%). Four genes (*TEM*, *the CTX-M-9 group*, *AIM*, and *NDM*) cause resistance to β-lactams; three genes (*aadA*, *strA*, *and strB*), to aminoglycosides; two genes (*sul2* and *sul3*), to sulfonamides; one gene (*fosA*), to fosfomycin; and one gene (*tetA*), to tetracyclines. Moreover, the most common resistance gene combination was *tetA-strB-sul2-TEM-NDM-strA-fosA-AIM-sul3-aadA-CTX-M-9*, detected in 67.2% of all isolates collected in 2014 and 2018.

Resistance genes *CTX-M-8*, *CTX-M-25*, *SIM*, *DIM*, and *GIM* were not found in any of the isolates. *SHV* and *SPM* were found only in three and two isolates, respectively, collected in 2018.

A comparison of the resistance genes detected in isolates from 2014 and 2018 showed that their frequencies changed over the five-year period. Three genes (*KPC*, *VIM* and *aac3(IV)*) were more common in 2018 than in 2014 (Table 2). Conversely, three genes (*CTX-M-1*, *tetC*, and *mrc-1*) were more common in 2014 than in 2018. The frequency of three genes (*TEM*, *strA*, and *tetA*) remained almost constant.

### 3.2. Identification of Virulence Genes in E. coli 

Virulence genes were analyzed in all isolates collected in 2014 and 2018. Thirteen virulence genes from four groups common to bloodstream-associated *E. coli* infections (toxins, serum resistance factors, iron uptake factors (siderophores), and adhesins) were tested (Table 3). The most common virulence genes were *fimH* (98.4% of all isolates), *fyuA* (91.8%), and *traT* (81.3%), which are responsible for type 1 fimbria adhesin activation, siderophore (yersiniabactin) activation, and bacterial serum resistance, respectively. The least common virulence gene was *ibeA*, which is responsible for activating adhesion group virulence factors. This gene was detected in only 17 isolates (6.6%, *p* < 0.05), which were mostly collected from healthcare institution II. Seven of these seventeen isolates were from 2014. The most common virulence gene combination was *fuyA-fimH-iroN*, found in 150 isolates (58.6%). *fuyA* and *iroN* encode siderophores, and *fimH* is responsible for bacterial adhesion to host cells.

Next, the virulence gene analysis results from 2014 and 2018 were compared to determine how the virulence determinants changed over time. The distribution of the virulence genes changed over the five-year period; some virulence genes increased in frequency, and others decreased (*p* value < 0.05) (Table 3). The frequency of three virulence genes (*hlyA*, *cnf1* and *sfaD/E*) increased from 2014 to 2018, whereas that of four genes (*cvaC*, *ibeA*, *papC* and *afaB/C*) decreased. 

### 3.3. E. coli Phylogenetic Classification

Phylogenetic group determination was performed for all isolates. The isolates belonged to four phylogenetic groups: A, B1, B2, and F. Group A isolates were detected at a significantly higher frequency (79.3% of all isolates, *p* < 0.05) than isolates of groups B1, B2, and F (Table 4). Group B1 isolates were the least common, with statistical significance (0.8%, *p* < 0.05), and included isolates from 2014 only.

Comparison of the phylogenetic group distribution in 2014 and 2018 showed that groups B2 and F were more common in 2018, whereas group A was more common in 2014 (Table 4). 

A dendrogram was constructed for the most common phylogenetic group, group A (Supplementary Figure S2). All group A isolates were separated into three groups according to the presence of phylogenetic genes: *yjaA* and *arpA* (only isolates 1598/2014 and 2142/2018) were detected in the first and second groups, respectively, whereas no genes were detected via quadruplex PCR in the third group. The most common virulence genes in phylogenetic group A were *fimH* (98.5% of phylogroup A isolates) and *fuyA* (89% of phylogroup A isolates). The most common virulence gene combination was *fimH-fyuA-traT* (73.4% of phylogroup A isolates). Moreover, the most common resistance genes were: *tetA* (100% of phylogroup A isolates), *strB* (99.5%), *sul2* (99.5%), *TEM* (99%), and *NDM* (98.5%); the most common resistance gene combination was *tetA-strB-sul2-TEM-NDM-strA-AIM-fosA-sul3-CTX-M-9-aadA-IMP* (62.1% of phylogroup A isolates). 

Moreover, in phylogenetic group B2 (Supplementary Figure S3), the most common virulence genes were *fyuA* (100% of phylogroup B2 isolates) and *fimH* (97.5% of phylogroup B2 isolates). The most common virulence gene combination was *fyuA-fimH-traT-sat* (60% of phylogroup B2 isolates). The most common resistance genes were *fosA* (100% of phylogroup B2 isolates), *sul2* (100%), *strA* (100%), and *tetA* (100%); the most common resistance gene combination was *fosA-sul2-strA-tetA-TEM-AIM-NDM-strB-aadA* (85% of phylogroup B2 isolates).

All isolates of phylogenetic group F (Supplementary Figure S4) had the virulence genes *fimH* and *traT*. There was no significant difference among phylogroup F isolates with respect to antibiotic resistance. 

### 3.4. rep-PCR Genotyping Results

The 256 tested isolates exhibited some genetic diversity, as revealed by rep-PCR methods. A total of 235 BOX-PCR profiles were established (Supplementary Figure S5). Dendrograms were constructed using UPGMA, and all profiles were separated into fourteen genotypic clusters. Cluster analysis showed that each cluster contained profiles with a variety of virulence and resistance genes; the presence of certain genes was not restricted to any specific cluster. 

Fourteen genotypic clusters were detected. Cluster 9 had the most isolates (*n* = 52, 20.3% of all isolates), and cluster 5 had the fewest (*n* = 1, 0.4%). Most of the isolates in cluster 9, the dominant cluster, were collected in 2018 (39 isolates), with fewer isolates collected in 2014 (17 isolates). Only cluster 8 had more isolates from 2014 (18 isolates) than from 2018 (13 isolates). All clusters included isolates from both years of the study. 

Cluster 1 lacked virulence genes *afaB/C* and *ibeA*, both of which are responsible for pathogenic *E. coli* adhesion and invasion of host cells. Clusters 5 and 8 lacked the antibiotic resistance genes *CTX-M-2* (β-lactam resistance) and *mrc-1* (colistin resistance), whereas cluster 7 lacked *tetB* (tetracycline resistance). 

**Figure 1:**
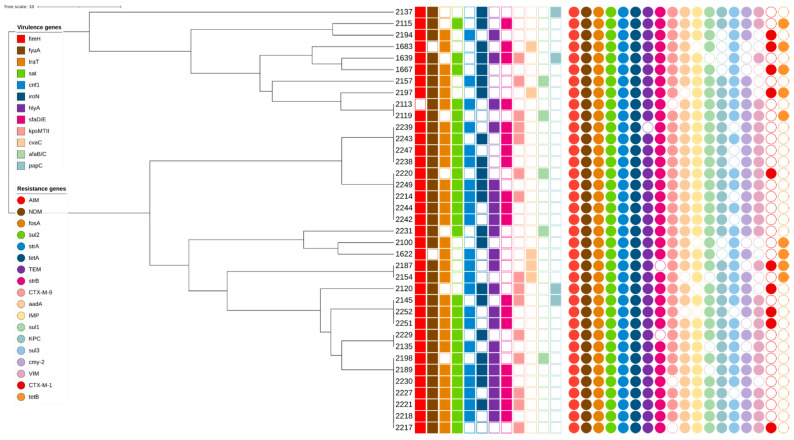
(GTG)5-PCR dendrogram.

Seventeen BOX-PCR profiles each contained two or three isolates. These profiles were distributed among clusters 1, 9, 10, 12, 13 and 14. No profiles with more than one isolate were found in clusters 2 and 3. All isolates were collected from different healthcare institutions (I, II, III, IV, V, VI, VII, VIII, IX, X, XI, and XII). (GTG)_5_-PCR analysis indicated that all analyzed isolates were genetically similar; only one isolate, 2137 (hospital XII), was different (Figure 1). The isolates were classified into two phylogenetic groups: A (29 isolates) and B2 (8 isolates). All isolates had different virulence gene profiles (Figure 1). Virulence genes *fimH* and *fyuA* were observed in all analyzed isolates except 1683 and 2113, respectively. All isolates also had different resistance gene profiles and gene combinations. The genes *aim*, *fosA*, *sul2*, *strA*, and *tetA*, which cause resistance to different types of antibiotic groups, were found in all isolates.

## 4. Discussion

This study was designed to characterize multidrug-resistant, bacteremia-causing *E. coli* isolates collected from different healthcare institutions in Lithuania in 2014 and 2018. Several techniques were used to characterize the isolates, namely, resistance and virulence gene determination, phylogenetic group detection, and rep-PCR genotyping, which are important methods for determining inter- and intra-hospital spread. 

The most common resistance gene was *tetA*, responsible for resistance to tetracyclines (observed in all isolates). Domínguez et al. [26] observed that *tetA*, which encodes an efflux mechanism, is the most common tetracycline resistance gene in *E. coli* in humans. The most common aminoglycoside resistance genes were *aadA* (92.6% of isolates), *strA* (98.4%), and *strB* (99.2%). These genes are responsible for the production of aminoglycoside-modifying enzymes (*strA*: adenyltransferase and phosphotransferases; *strB*; phosphotransferases), which leads to antibiotic deactivation by modification [27]. 

β-lactam resistance genes were also observed. The most common β-lactamase gene was *TEM* (98.8% of all isolates). In comparison, in our previous study [10], we observed a lower frequency of this gene (33.9%). *CTX-M-9* was the most commonly detected *CTX-M* group gene (90.6% of all isolates). This result differs from those of previous studies in Lithuania and northern countries [28]; *CTX-M-9* was not observed among Lithuanian clinical isolates, and the most common gene in the *CTX-M* group was *CTX-M-15*. This difference suggests that this gene emerged in Lithuania between 2012 (when the isolates analyzed by Sepp et al. were collected) and 2014 (when isolates were collected for the present study). Furthermore, we observed a high frequency of two additional metallo-β-lactamase genes in this study: *NDM*, which was previously detected in Lithuania [10], and *AIM*, which was not, were detected in 98.4% and 97.3% of all isolates, respectively. 

One virulence mechanism responsible for the survival of *E. coli* strains in the bloodstream is the escape of recognition by the complement system through serum resistance [4]. Multiple virulence factors are involved in serum survival. In this study, 81.3% of isolates had the *traT* gene. Similar results were found in studies of *E. coli* bloodstream infections worldwide [29,30,31]. *E. coli* capsule research showed that almost half (49.2%) of all isolates expressed *kpsMTII*, which codes for K1 and K5 capsules. These results confirm previous research on bloodstream infections [32]. 

Siderophores represent another virulence mechanism by which pathogenic bacteria take up iron from host molecules. Siderophores are commonly associated with pathogenic *E. coli* strains isolated from bacteremia cases [29,31]. In this study, yersiniabactin (*fyuA*) was the most prevalent siderophore, present in 91.8% of the tested isolates. This *fyuA* gene product is involved in the efficient uptake of iron from the bloodstream [21] and the bacterial invasion of the bloodstream from the urinary tract [18]. Another siderophore, salmocherin (*iroN*), is less common (present in 62.5% of isolates) and is mostly associated with invasion of the bloodstream from the urinary tract [18]. 

Virulence genes related to adhesins were also investigated during this study, including type 1 fimbriae (*fimH*), S fimbria (*staD/E*), P fimbria (*papC*), and Dr binding adhesin (*afaB/C*). The most common gene was *fimH* (98.4%), which is a critical virulence factor of uropathogenic *E. coli* strains, facilitating adhesion to uroepithelial proteins [7]. Bloodstream infections usually occur as a complication of the urinary tract infections promoted by this factor [9]. 

The virulence genes related to toxin and hemolysin production observed in this study included *sat*, *ibeA*, *cvaC*, *hlyA*, and *cnf1*. The most common was *sat* (53.3% of all isolates), which encodes secreted autotransporter toxin. This toxin has proteolytic activity and influences the vacuolization of urinary epithelial cells [33]. As a result, it is mostly associated with bacteremic urinary tract infection. The second-most common toxin-encoding gene was *hlyA* (40.3% of all isolates), which encodes α-hemolysin, a pore-forming bacterial exotoxin that may contribute to the virulence of bacteria during bloodstream infections and sepsis [34]. 

According to the phylogenetic group classification of the *E. coli* isolates, group A was the most common (79.3%). In the literature, B2 was reported to be the most common phylogenetic group of pathogenic *E. coli* [4,29], and this was corroborated by the results obtained in previous studies in Lithuania [35,36]. However, in our study, the B2 phylogroup comprised only 15.6% of all isolates, mostly those collected in 2018 (10 isolates from 2014 and 30 isolates from 2018). The same phylogenetic group pattern was observed in Romania, where group A was the dominant group and group B2 was less prevalent [37]. According to that data, isolates of group A are usually strictly commensal strains found in the intestinal microbiota. Another study conducted by Fratamico et al. [38] reported that isolates of groups B2 and D have a higher virulence in humans, allowing them to induce extraintestinal infections in both healthy and immunocompromised hosts. However, an old classification system established by Clermont et al. [22] was used in that study, and there was no distinction between groups D, E, and F; all strains were classified as group D. Conversely, in our study, a newer classification system [5] was used, and group D isolates were absent. However, group F isolates comprised 4.3% of all isolates. They contained virulence genes *fimH* and *traT*, which are responsible for effective *E. coli* adhesion and host serum resistance, respectively. 

Based on the BOX-PCR genotyping results, the isolates were divided into fourteen clusters. The most dominant cluster was cluster 9 (20.3%). Cluster analysis showed that all isolates were genetically very similar, with no significant differences associated with collection year or healthcare institution. Furthermore, the genetic profiles of isolates in each cluster contained a variety of virulence and resistance genes; the presence of certain genes was not restricted to any specific cluster. However, in cluster 9, isolates collected in 2018 were more prevalent than those collected in 2014. A possible explanation for these results could be the successful genetic adaptation of bacteria over time and the dissemination of resistance genes, which can occur through transmission via medical staff, contaminated equipment, or patients transferring between healthcare institutions [39]. These data are similar to our previously reported results indicating that clinical isolates of *E. coli* are spread via intra- and/or inter-hospital dissemination between Lithuanian healthcare institutions [10]. Moreover, 17 BOX-PCR profiles each contained two or three isolates. Further analysis via (GTG)_5_-PCR showed that all analyzed isolates were genetically very similar; only one isolate, from hospital XII (2137), had a significantly different genetic profile. Moreover, a detailed analysis of the 17 BOX-PCR profiles showed that two virulence genes, *fimH* and *fyuA*, were observed in almost all isolates. Resistance genes found in all isolates included *AIM*, *fosA*, *sul2*, *strA*, and *tetA*, which are responsible for different types of antibiotic resistance mechanisms. 

In summary, the study results indicated that the multidrug resistance of bacteremia-causing *E. coli* is induced by various combinations of different genetic determinants. The variety of resistance and virulence genes can be explained by genetic plasticity determinants, such as plasmids and transposable elements, and other factors, such as inter- and intra-hospital transmission. Furthermore, 235 BOX-PCR profiles were established, and all isolates exhibited relatively similar genotyping results. However, more studies are needed to evaluate the intra- and inter-hospital transmission of *E. coli* isolates and analyze their adaptations to antibiotic strategies.

## Figures and Tables

**Table 1:** Characterization of genes associated with antibiotic resistance and virulence.

Gene		Reference to Gene Primers
Antibiotic Resistance Genes	Resistance Mechanism	
*blaNDM*	β-lactams resistance genes	[11]
*blaKPC*		
*Multiplex I TEM*		[12]
*Multiplex SHV*		
*Multiplex II CTX-M group 1*		
*Multiplex II CTX-M group 2*		
*Multiplex II CTX-M group 9*		
*Multiplex III CTX-M group 8/25*		
*blaCMY-2*		[13]
*blaIMP*		[14]
*blaVIM*		
*blaSPM*		
*blaAIM*		
*blaGIM*		
*blaSIM*		
*blaDIM*		
*fosA*	Fosfomycin resistance gene (glutathione S-transferase)	[15]
*aadA*	Aminoglycoside resistance genes	[16]
*strA*		
*strB*		
*aac(3)IV*		
*tetA*	Tetracycline resistance genes	[16]
*tetB*		
*tetC*		
*sul1*	Sulphonamide resistance genes	[16]
*sul2*		
*sul3*		
*mcr-1*	Colistin resistance gene	[17]
Virulence genes	Virulence mechanism	
Siderophores		
*iroN*	salmocherin	[18]
*fyuA*	yersiniabactin	
Adhesins		
*sfaD/E*	S fimbriae	[19]
*papC*	P fimbriae	
*afaB/C*	Dr binding adhesin	
*fimH*	type 1 fimbriae	[18]
Toxins		
*hlyA*	α-hemolysin	[20]
*cnf1*	necrotizing cytotoxic factor type 1	
*sat*	autotransporter toxin	[21]
*cvaC*	colicin V	[18]
*ibeA*	Ibe A cell invasin	
Serum resistance		
*kpsMTII*	K1 and K5 capsules	[18]
*traT*	complement resistance protein	

**Table 2:** Distribution of different antibiotic resistance genes in 2014 and 2018.

Resistance Genes Group	Resistance Gene	Total Number of Isolates (*n* = 256), *n* (%)	Number of Isolates in 2014 (*n* = 91), *n* (%)	Number of Isolates in 2018 (*n* = 165), *n* (%)	% of Increase (+%)/or Decrease (−%)	*p* Value
β-lactams resistance genes	*NDM*	252 (98.4%)	88 (96.7%)	164 (99.4%)	+2.7%	>0.05
	*KPC*	186 (72.7%)	37 (40.7%)	149 (90.3%)	+49.6%	0.00002
	*TEM*	253 (98.8%)	90 (98.9%)	163 (98.8%)	+0.1%	>0.05
	*AIM*	249 (97.3%)	89 (97.8%)	160 (96.7%)	−1.1%	>0.05
	*CTX-M-1*	87 (34%)	45 (49.5%)	42 (25.5%)	−24%	0.009
	*CTX-M-2*	35 (13.7%)	11 (12.1%)	24 (14.5%)	+2.4%	>0.05
	*CTX-M-9*	232 (90.6%)	90 (98.9%)	142 (86.1%)	−12.8%	>0.05
	*IMP*	227 (88.7%)	77 (84.6%)	150 (90.9%)	+6.3%	>0.05
	*VIM*	134 (52.3%)	36 (39.5%)	98 (59.4%)	+19.9%	0.050
	*CMY-2*	183 (71.5%)	54 (59.3%)	129 (78.2%)	+18.9%	>0.05
Sulphonamide resistance genes	*sul1*	198 (77.3%)	63 (69.2%)	135 (81.8%)	+12.6%	>0.05
	*sul2*	254 (99.2%)	90 (98.9%)	164 (99.4%)	+0.5%	>0.05
	*sul3*	237 (92.6%)	91 (100%)	146 (88.5%)	−11.5%	>0.05
Aminoglycoside resistance genes	*strA*	252 (97.7%)	90 (98.9%)	162 (98.2%)	−0.7%	>0.05
	*strB*	254 (99.2%)	91 (100%)	163 (98.8%)	−1.2%	>0.05
	*aac3 (IV)*	45 (17.6%)	9 (9.9%)	36 (21.8%)	+11.9%	0.037
	*aadA*	237 (92.6%)	82 (90.1%)	155 (93.8%)	+3.7%	>0.05
Tetracycline resistance genes	*tetA*	256 (100%)	91 (100%)	165 (100%)	0	>0.05
	*tetB*	53 (20.7%)	23 (25.3%)	30 (18.2%)	−7.1%	>0.05
	*tetC*	38 (14.8%)	23 (25.3%)	15 (9.1%)	−16.2%	0.0009
Fosfomycin resistance gene	*fosA*	249 (97.3%)	88 (96.7%)	161 (97.6%)	+0.9%	>0.05
Colistin resistance gene	*mrc-1*	13 (5.1%)	8 (8.8%)	5 (3.03%)	−5.77%	0.043

**Table 3:** Distribution of virulence genes at different healthcare institutions.

Virulence Factor	Genes	Number of Isolates in Healthcare Institutions (*n* = 256), *n* (%)	Number of Isolates in 2014 (*n* = 91), *n* (%)	Number of Isolates in 2018 (*n* = 165), *n* (%)	% of Increase (+%)/or Decrease (−%)	*p* Value
Toxins	*hlyA*	104 (40.6%)	21 (23.1%)	83 (50.3%)	+27.2%	0.004
	*cnf1*	86 (33.6%)	16 (17.6%)	70 (42.2%)	+24.6%	0.003
	*cvaC*	46 (18%)	23 (25.3%)	23 (13.9%)	−11.4%	0.018
	*ibeA*	17 (6.6%)	10 (11%)	7 (4.2%)	−6.8%	0.027
	*sat*	138 (53.9%)	40 (44%)	98 (59.4%)	+15.4	>0.050
Adhesins	*staD/E*	122 (47.7%)	31 (34.1%)	91 (55.2%)	+21.1	0.05
	*fimH*	252 (98.4%)	89 (97.8%)	163 (98.8%)	+1%	>0.05
	*papC*	47 (18.4%)	28 (30.8%)	19 (11.5%)	−19.3%	0.0002
	*afaB/C*	34 (13.3%)	19 (20.9%)	15 (9.1%)	−11.8%	0.006
Siderophores	*fyuA*	235 (91.8%)	81 (89%)	154 (93.3%)	+4.3%	>0.05
	*iroN*	161 (62.9%)	54 (59.3%)	107 (64.8%)	+5.5%	>0.05
Serum resistance	*traT*	208 (81.3%)	69 (75.8%)	139 (84.2%)	+8.4%	>0.05
	*kpsMTII*	126 (49.2%)	47 (51.6%)	79 (47.9%)	−3.7%	>0.05

**Table 4:** Phylogenetic groups detected in 2014 and 2018.

	Phylogenetic Group A	Phylogenetic Group B1	Phylogenetic Group B2	Phylogenetic Group F
Total number of isolates (*n* = 256), *n*/%	203/79.3%	2/0.8%	40/15.6%	11/4.3%
Number of isolates in 2014 (*n* = 91), *n*/%	77/84.6%	2/2.2%	10/11%	2/2.2%
Number of isolates in 2018 (*n* = 165), *n*/%	126/76.4%	-	30/18.2%	9/5.5%

## Data Availability

Not applicable.

## Supplementary Materials

The following are available online at https://www.mdpi.com/article/10.3390/microorganisms10020449/s1, Table S1: Collected isolates. Figure S1: Dendrogram of phylogenetic group A. Figure S2: Dendrogram of phylogenetic group B2. Figure S3: Dendrogram of phylogenetic group F. Figure S4: BOX-PCR dendrogram.
