# Supplementary material for: Genetic Characterization of Multidrug-Resistant E. coli Isolates from Bloodstream Infections in Lithuania
Source: Microorganisms. 2022 Feb 15;10(2):449. doi: 10.3390/microorganisms10020449 (PMC8880137; doi:10.3390/microorganisms10020449)
Supplement: Supplementary file 1 [file microorganisms-10-00449-s001.zip › Supplementary Figure S2. Dendrogram of phylogenetic group B2..pdf]

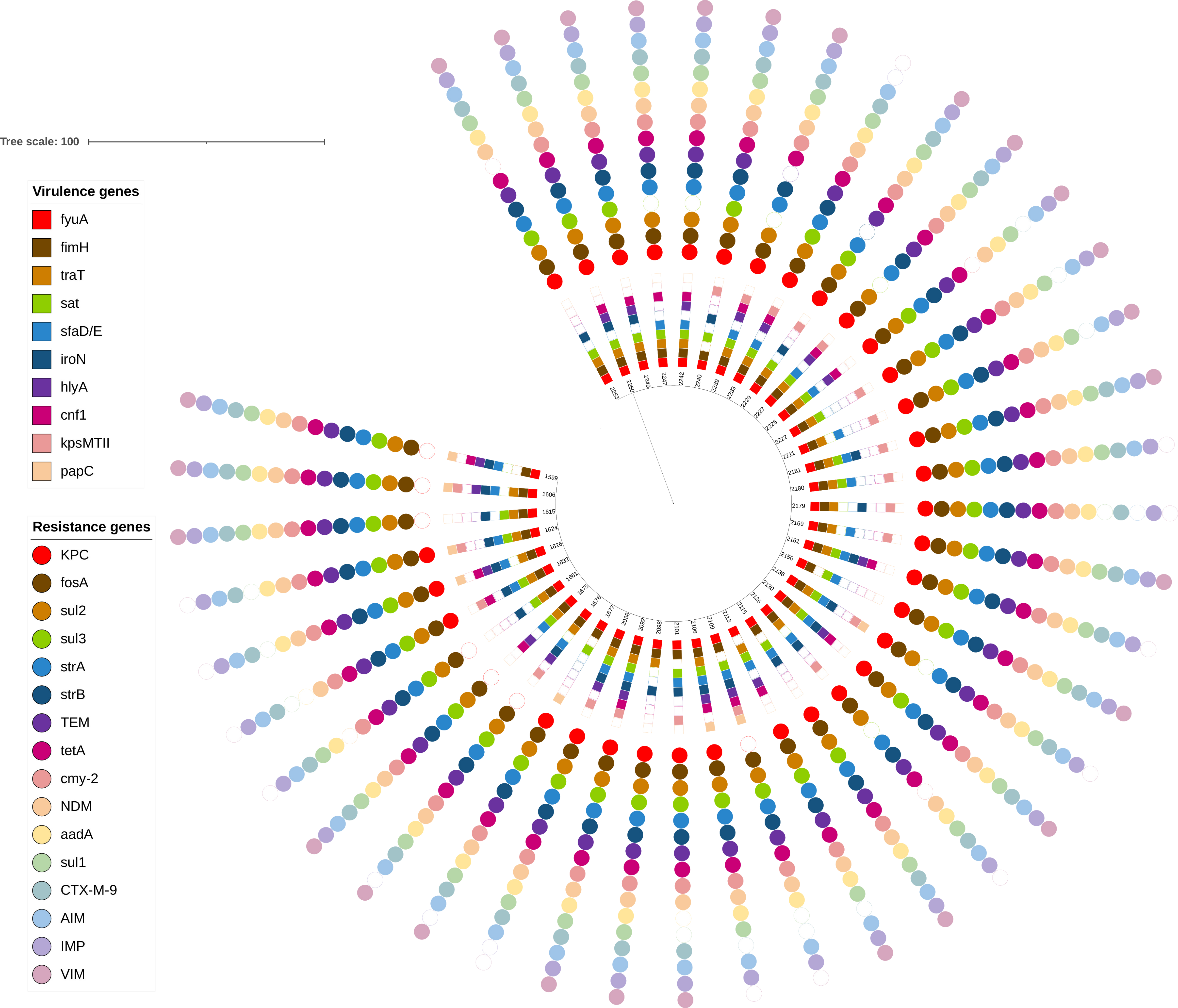
Tree scale: 100
Virulence genes
fyuA
fimH
traT
sat
sfaD/E
iroN
hlyA
cnf1
kpsMTII
papC
Resistance genes
KPC
fosA
sul2
sul3
strA
strB
TEM
tetA
cmy-2
NDM
aadA
sul1
CTX-M-9
AIM
IMP
VIM
1599
1606
1615
1624
1626
1632
1661
1675
1676
1677
2088
2092
2098
2101
2106
2109
2113
2115
2126
2130
2136
2156
2161
2169
2179
2180
2181
2211
2222
2225
2227
2229
2233
2239
2240
2242
2247
2249
2250
2253
