# Supplementary material for: Genetic Characterization of Multidrug-Resistant E. coli Isolates from Bloodstream Infections in Lithuania
Source: Microorganisms. 2022 Feb 15;10(2):449. doi: 10.3390/microorganisms10020449 (PMC8880137; doi:10.3390/microorganisms10020449)
Supplement: Supplementary file 1 [file microorganisms-10-00449-s001.zip › Supplementary Table S1. Collected isolates..pdf]

Isolate	Year of isolation	Healthcare institution
20141593	2014	X
20141594	2014	II
20141595	2014	IV
20141596	2014	II
20141597	2014	XI
20141598	2014	XI
20141599	2014	II
20141600	2014	X
20141601	2014	I
20141602	2014	II
20141603	2014	IV
20141604	2014	I
20141605	2014	II
20141606	2014	IV
20141607	2014	I
20141608	2014	I
20141609	2014	I
20141610	2014	VII
20141611	2014	I
20141612	2014	V
20141613	2014	VI
20141614	2014	V
20141615	2014	V
20141616	2014	VI
20141617	2014	II
20141618	2014	V
20141619	2014	VII
20141620	2014	VIII
20141621	2014	VIII
20141622	2014	VII
20141623	2014	II
20141624	2014	II
20141625	2014	II
20141626	2014	II
20141627	2014	I
20141628	2014	I
20141629	2014	II
20141630	2014	I
20141631	2014	X
20141632	2014	II
20141633	2014	VII
20141634	2014	XI
20141635	2014	IV
20141636	2014	VII
20141637	2014	VI
20141638	2014	II
20141639	2014	II
20141640	2014	IV

20141641	2014	IV
20141642	2014	II
20141643	2014	IX
20141644	2014	VIII
20141645	2014	VIII
20141646	2014	VIII
20141647	2014	VIII
20141648	2014	VIII
20141649	2014	VIII
20141650	2014	VIII
20141651	2014	X
20141652	2014	II
20141653	2014	II
20141654	2014	II
20141655	2014	II
20141656	2014	II
20141657	2014	II
20141658	2014	II
20141659	2014	II
20141660	2014	XI
20141661	2014	VI
20141662	2014	XI
20141663	2014	II
20141664	2014	II
20141665	2014	X
20141666	2014	IV
20141667	2014	VII
20141668	2014	V
20141669	2014	V
20141670	2014	II
20141671	2014	II
20141672	2014	II
20141673	2014	I
20141674	2014	II
20141675	2014	I
20141676	2014	II
20141677	2014	I
20141678	2014	IV
20141679	2014	V
20141680	2014	II
20141681	2014	II
20141682	2014	VI
20141683	2014	II
20182088	2018	VIII
20182089	2018	VIII
20182090	2018	IV
20182091	2018	V
20182092	2018	V
20182093	2018	III

20182094	2018	XII
20182095	2018	I
20182096	2018	III
20182097	2018	IV
20182098	2018	XII
20182099	2018	VIII
20182100	2018	VI
20182101	2018	VI
20182102	2018	II
20182103	2018	II
20182104	2018	VI
20182105	2018	V
20182106	2018	II
20182107	2018	II
20182108	2018	II
20182109	2018	II
20182110	2018	II
20182111	2018	II
20182112	2018	II
20182113	2018	I
20182114	2018	XI
20182115	2018	X
20182116	2018	II
20182117	2018	VI
20182118	2018	VI
20182119	2018	V
20182120	2018	II
20182121	2018	V
20182122	2018	II
20182123	2018	X
20182124	2018	I
20182125	2018	V
20182126	2018	I
20182127	2018	X
20182128	2018	I
20182129	2018	XII
20182130	2018	X
20182131	2018	I
20182132	2018	I
20182133	2018	X
20182134	2018	III
20182135	2018	VI
20182136	2018	X
20182137	2018	XII
20182138	2018	VIII
20182139	2018	IV
20182140	2018	VIII
20182141	2018	IX
20182142	2018	I

20182143	2018	VIII
20182144	2018	VIII
20182145	2018	IV
20182146	2018	X
20182147	2018	VIII
20182148	2018	VIII
20182149	2018	VIII
20182150	2018	I
20182151	2018	VIII
20182152	2018	VIII
20182153	2018	IX
20182154	2018	I
20182155	2018	X
20182156	2018	III
20182157	2018	VI
20182158	2018	I
20182159	2018	X
20182160	2018	IX
20182161	2018	XI
20182162	2018	VIII
20182163	2018	VII
20182164	2018	I
20182165	2018	VIII
20182166	2018	VIII
20182167	2018	IX
20182168	2018	XI
20182169	2018	V
20182170	2018	V
20182171	2018	V
20182172	2018	V
20182173	2018	V
20182174	2018	VIII
20182175	2018	V
20182176	2018	VI
20182177	2018	XI
20182178	2018	VIII
20182179	2018	VIII
20182180	2018	X
20182181	2018	VI
20182182	2018	VIII
20182183	2018	I
20182184	2018	VIII
20182185	2018	IV
20182186	2018	VI
20182187	2018	I
20182188	2018	XI
20182189	2018	X
20182190	2018	I
20182191	2018	VIII

20182192	2018	IV
20182193	2018	VIII
20182194	2018	V
20182197	2018	VII
20182198	2018	III
20182199	2018	X
20182200	2018	VIII
20182201	2018	X
20182202	2018	VI
20182203	2018	III
20182204	2018	II
20182205	2018	VIII
20182206	2018	VIII
20182207	2018	VIII
20182208	2018	VIII
20182209	2018	VIII
20182210	2018	VIII
20182211	2018	IV
20182212	2018	IV
20182213	2018	VIII
20182214	2018	I
20182215	2018	IX
20182216	2018	I
20182217	2018	VII
20182218	2018	XI
20182219	2018	XI
20182220	2018	X
20182221	2018	III
20182222	2018	VIII
20182223	2018	XII
20182224	2018	I
20182225	2018	X
20182226	2018	VI
20182227	2018	I
20182228	2018	VIII
20182229	2018	VIII
20182230	2018	I
20182231	2018	IX
20182232	2018	I
20182233	2018	IX
20182234	2018	X
20182235	2018	I
20182236	2018	X
20182237	2018	IV
20182238	2018	I
20182239	2018	I
20182240	2018	VI
20182241	2018	VIII
20182242	2018	IX

20182243	2018	IV
20182244	2018	VIII
20182245	2018	VIII
20182246	2018	IX
20182247	2018	V
20182248	2018	V
20182249	2018	XI
20182250	2018	XII
20182251	2018	VI
20182252	2018	XI
20182253	2018	I
20182254	2018	I
