# Supplementary figures and images for: Genetic Characterization of Multidrug-Resistant E. coli Isolates from Bloodstream Infections in Lithuania
Source: Microorganisms. 2022 Feb 15;10(2):449. doi: 10.3390/microorganisms10020449 (PMC8880137; doi:10.3390/microorganisms10020449)

Tree scale: 0.1

### Virulence genes

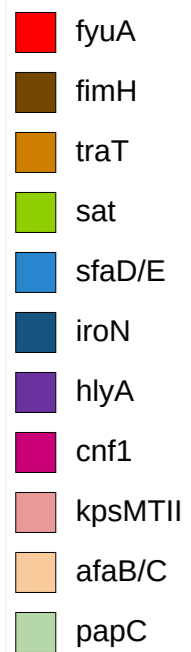

### Resistance genes

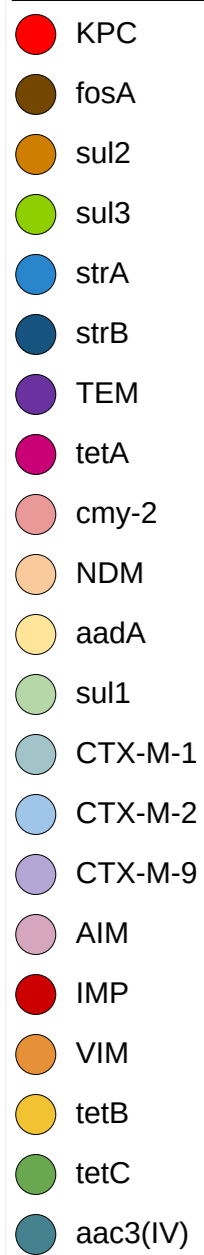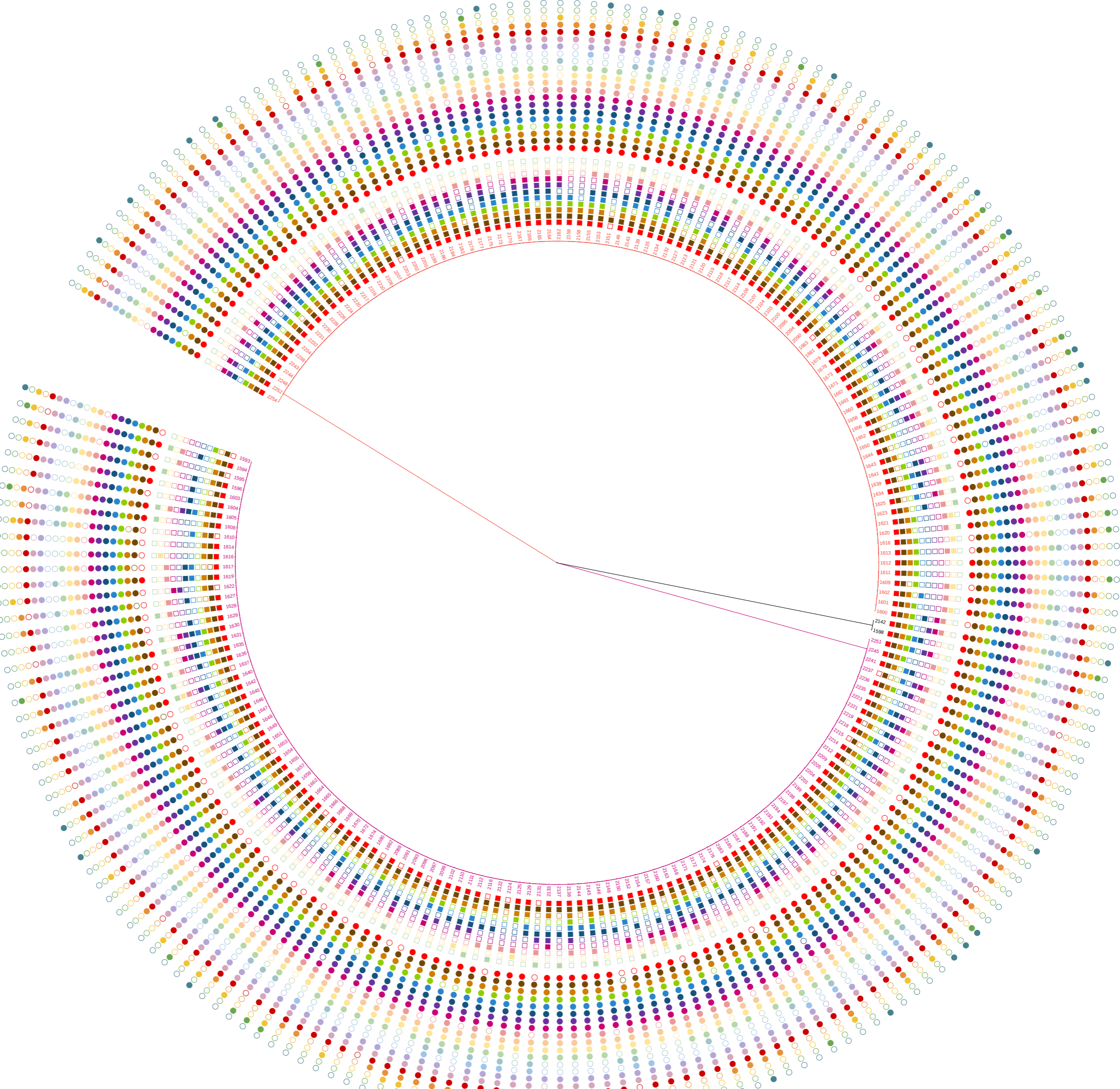

Supplement: Supplementary file 1 [file microorganisms-10-00449-s001.zip › Supplementary Figure S1. Dendrogram of phylogenetic group A..pdf]

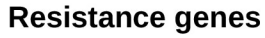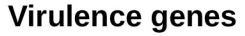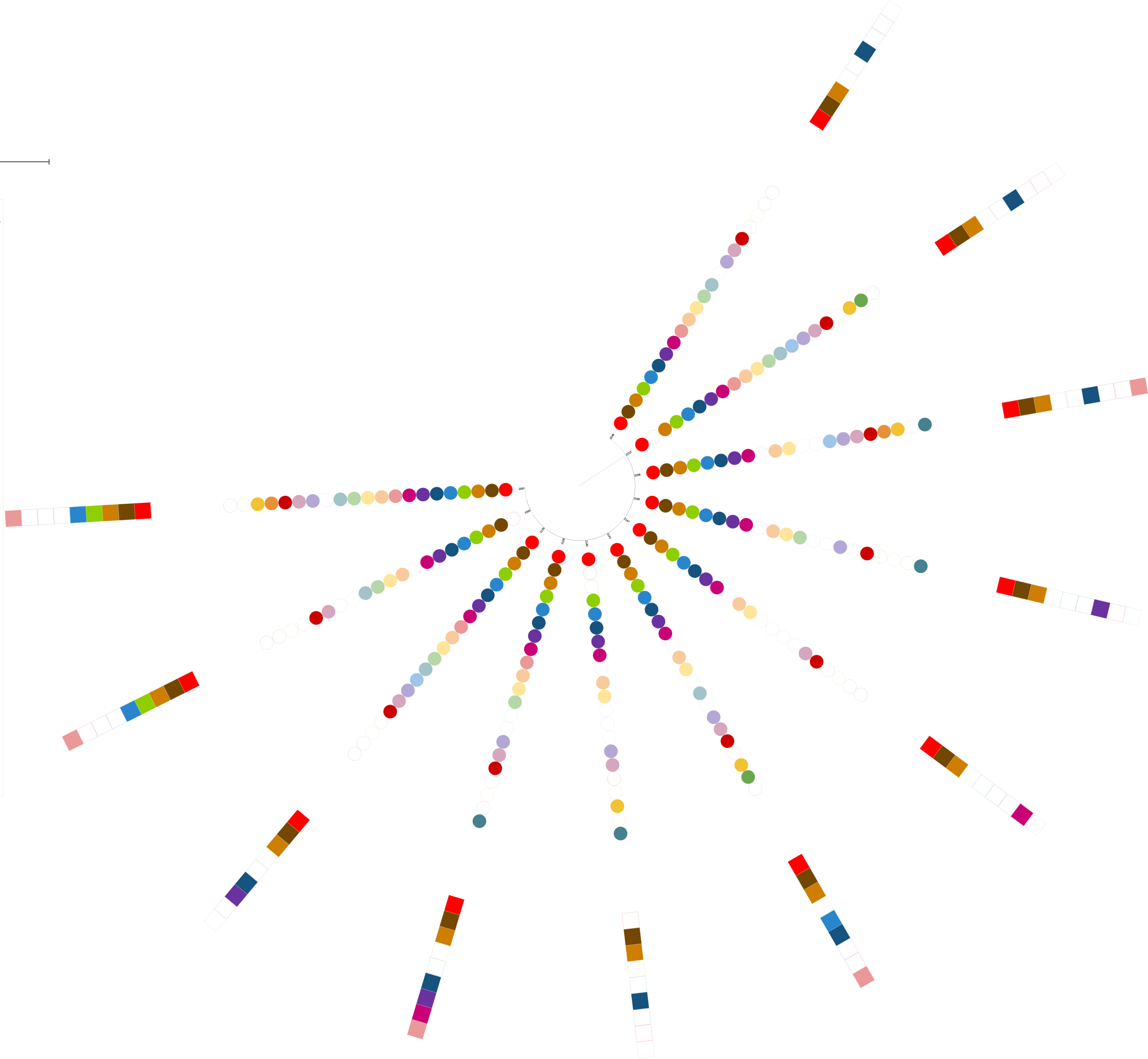

Supplement: Supplementary file 1 [file microorganisms-10-00449-s001.zip › Supplementary Figure S3. Dendrogram of phylogenetic group F..pdf]

Tree scale: 1

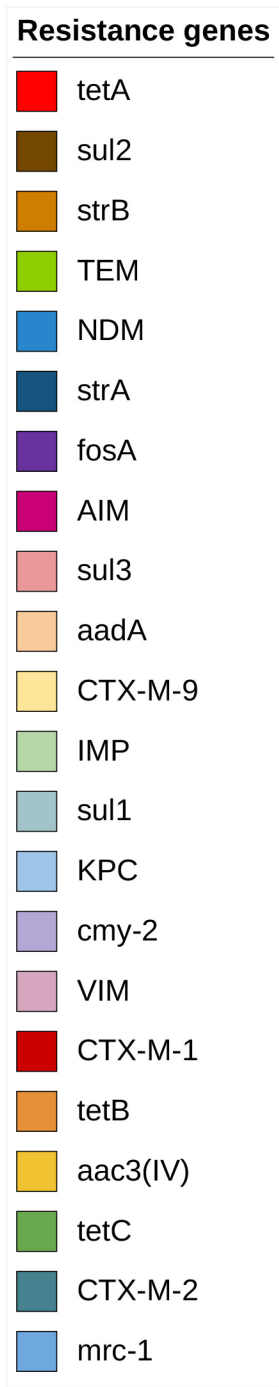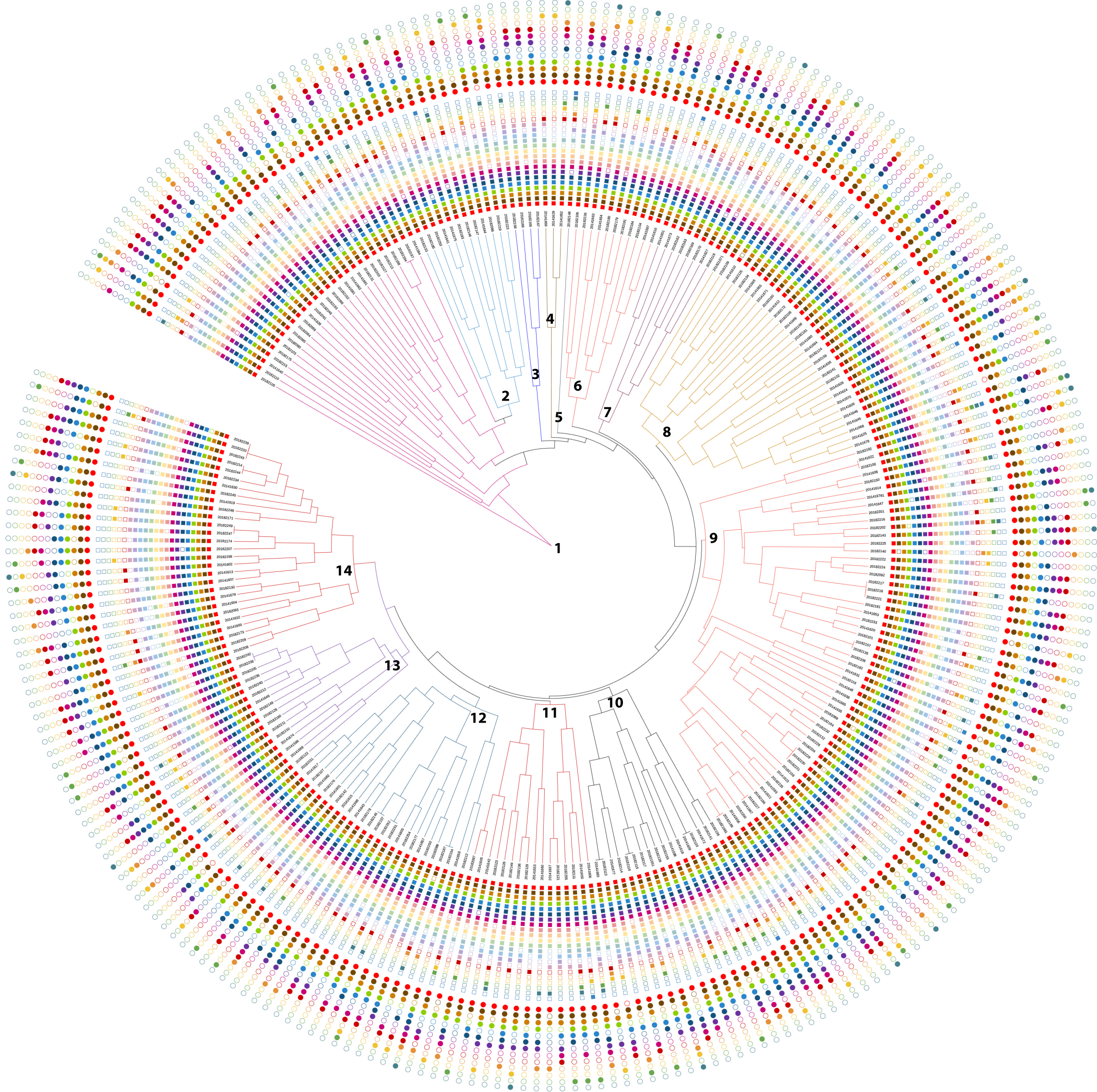

Supplement: Supplementary file 1 [file microorganisms-10-00449-s001.zip › Supplementary Figure S4. BOX-PCR dendrogram..pdf]
